# Ethics in editorship: lessons from a major retraction incident

**DOI:** 10.1038/s41431-025-01847-4

**Published:** 2025-04-09

**Authors:** Joris A. Veltman

**Affiliations:** Institute of Genetics and Cancer, College of Medicine and Veterinary Medicine, University of Edinburgh, Edinburgh, UK

**Keywords:** Genetic variation, Ethics

Being asked to join the editorial board of a journal is a big honour, but it also comes with big responsibilities, especially in our field of human genetics. Here, I want to reflect on a serious incident involving the journal ‘Molecular Genetics and Genomic Medicine’ (MGGM) for which I served as an editorial board member. This journal was targeted in 2020 and 2021 by multiple Chinese institutions to publish genetic profiling studies of vulnerable populations. None of this was noted by us, the editorial board, during or after the publication of a total of 18 papers. Instead, concerns were raised by a colleague, Professor Yves Moreau, who opened our eyes to this issue. Eventually, it led to the largest set of retractions ever over human rights issues. Clearly, as editorial board members, we could and should have interfered much earlier. In today’s world, we cannot simply lean back and collect editorial board memberships as status symbols. Instead, we should regularly check what is being published in our journals and make sure publishers are aware of and responsive to the many sensitive ethical issues in human genetics.

So, what happened? On March 14, 2021, Yves Moreau, a computational biologist specializing in human genetics and a Professor of Engineering at the University of Leuven, contacted the Editor-in-Chief of MGGM and copied me into this email. He notified us that the journal had become the premier venue for the publication of forensic genetics research on vulnerable Tibetan and Muslim minorities from China. Initially, he pointed us to 13 publications, later extended to 18, published in MGGM in the last 18 months. Many of the publications involved authors affiliated with Chinese public security, the judiciary, or institutions directly under their control. Of note, forensic genetics or population genetics are not mentioned in the scope of MGGM, and it has remained unclear how these publications ended up in this journal and whether there was sufficient editorial expertise to evaluate these manuscripts prior to publication. Yves asked the Editor-in-Chief to initiate a full ethical reassessment of these publications, not only for compliance regarding informed consent but also against the standards of the Declaration of Helsinki regarding vulnerable groups and against ethical principles of benevolence, non-maleficence, and justice. Earlier that year, the EJHG published a policy statement from the European Society of Human Genetics, with Professor Moreau as a co-author, warning against misuse of genetic tests and biobanks for discrimination [1].

Unfortunately, there was no clear response from the Editor-in-Chief after repeated reminders from Yves and me, and on June 14, 2021, Yves involved the entire editorial board as well as the publisher Wiley to raise awareness of this issue and ask for action. Like me, other editorial board members were unaware of these publications and asked for a rapid response and action from the Editor-in-Chief and publisher. When this did not happen, 8 editorial board members resigned from their role, something that received a lot of attention in scientific and popular media outlets [2–4]. In contrast to these 8 colleagues, I decided that it was important to stay on the board to ensure proper investigation was carried out by the publisher and Editor-in-Chief. Eventually, Wiley informed us that they did take immediate action after receiving this information from Yves, and that they had begun a comprehensive investigation through their Integrity in Publishing Group, in accordance with the Committee on Publication Ethics (COPE) guidelines [5].

Clearly, such investigations are complex, especially when they involve not 1 but 18 publications. Yves and I regularly asked for updates from the publisher and tried to keep the pressure on, and so did other editorial board members, but we were largely kept in the dark and had to wait for almost 3 years. On February 14, 2024, Wiley finally retracted all 18 papers [6], again receiving a lot of attention in the media [7, 8]. Their investigation uncovered inconsistencies between the consent documentation and the research reported. The associated data for these articles was also withdrawn as the consent information did not give approval for data to be shared publicly and could be used to identify those who participated in the research.

While this mass retraction is a very important and positive decision by the publisher, many questions remain unanswered. Why did so many Chinese colleagues target this genetics journal for their forensic studies on vulnerable minorities? Who handled the review of these publications, and why did the journal review process not uncover any issues? Open and honest communication between the Editor-in-Chief, the editorial board and the publisher is paramount in this aspect, especially during difficult processes such as those outlined here. I did not feel this was handled well in MGGM and now that the papers are retracted, I have resigned from the editorial board.

Clearly, this is not the end to the misuse of genetic data, and MGGM is not the only journal in which these problematic studies are being published. As editors but also as reviewers for genetics journals, we should take our responsibilities very seriously and make sure that our journals adhere to ethical guidelines and principles. Of note, COPE aims to move the culture of publishing towards one where ethical practices become a normal part of the culture itself. It provides training and support for editors as well as publishers, but, of course, it does not specifically address genetic profiling studies. The ESHG also organizes a new virtual course entitled ‘Covering the Gaps’, which includes a discussion by Yves Moreau on the use and misuse of data. As geneticists, we should remain alert to these attempts and regularly remind ourselves that ethics is not a checkbox exercise [9].
